# Application of ecological momentary assessment in stress-related diseases

**DOI:** 10.1186/1751-0759-2-13

**Published:** 2008-07-11

**Authors:** Kazuhiro Yoshiuchi, Yoshiharu Yamamoto, Akira Akabayashi

**Affiliations:** 1 Department of Stress Sciences and Psychosomatic Medicine, Graduate School of Medicine, the University of Tokyo, Tokyo, Japan; 2 Educational Physiology Laboratory, Graduate School of Education, The University of Tokyo, Tokyo, Japan; 3 Department of Stress Sciences and Psychosomatic Medicine, Graduate School of Medicine, the University of Tokyo, 7-3-1 Hongo, Bunkyo-ku, Tokyo 113-8655, Japan

## Abstract

Many physical diseases have been reported to be associated with psychosocial factors. In these diseases, assessment relies mainly on subjective symptoms in natural settings. Therefore, it is important to assess symptoms and/or relationships between psychosocial factors and symptoms in natural settings. Symptoms are usually assessed by self-report when patients visit their doctors. However, self-report by recall has an intrinsic problem; "recall bias". Recently, ecological momentary assessment (EMA) has been proposed as a reliable method to assess and record events and subjective symptoms as well as physiological and behavioral variables in natural settings. Although EMA is a useful method to assess stress-related diseases, it has not been fully acknowledged, especially by clinicians. Therefore, the present brief review introduces the application and future direction of EMA for the assessment and intervention for stress-related diseases.

## Introduction

Many physical diseases have been reported to be associated with psychosocial factors such as irritable bowel syndrome (IBS) [1], primary headaches [2], and asthma [3]. In these diseases, assessment relies mainly on subjective symptoms in natural settings. Therefore, it is important to assess symptoms and/or relationships between psychosocial factors and symptoms in natural settings.

Symptoms are usually assessed by self-report when patients visit their doctors. Most self-reported data are collected by questionnaires or interviews that ask patients to summarize past symptoms over some period of time. For example, a pain questionnaire might ask about the intensity of last week's pain. However, self-report by recall has an intrinsic problem; "recall bias". Many research data have shown that people are not be able to accurately recall past experience, particularly experiences that are frequent, mundane, and irregular, because self-report data are affected by recall biases such as their mood states (state biases) (Table 1) [4]. In addition to the state biases, there are other recall biases affecting self-report data: (1) recency, which means that more recent events are more accessible to memory, (2) saliency, which means that salient experiences are more likely to be encoded and subsequently recalled, (3) effort after meaning, which means that people's natural and unconscious tendency is to reconstruct events so as to make them consistent with subsequent events, (4) participants' misunderstanding of questionnaire instruction sets that require them to aggregate and summarize their experience in the recent past, and (5) aggregation, which is cognitive processing that is necessary to respond to questions about the occurrence or frequency of events or about their average or typical characteristics [5]. In fact, there have been some studies that show inconsistency between recalled symptoms and momentary recorded symptoms [6-8]. Especially, previous studies indicated that variability of symptoms could affect the recall (Table 2) [7,8].

**Table 1:** Recall biases affecting self-report data

State biases
Recency
Saliencey
Effort after meaning
Misunderstanding of instruction set
Aggregation

**Table 2:** Consistency between recalled headache intensity and momentary headache intensity for the two subgroups of patients with tension-type headache

	low SD group		high SD group	
	
	Mean (SD)	ICC (A, 1) (95% C.I.)	Mean (SD)	ICC (A, 1) (95% C.I.)
Recalled headache intensity and	54.7 (22.5)		59.5 (17.8)	
mean headache intensity of all recordings	41.3 (23.2)*	0.75 (0.04, 0.93)	33.5 (16.8)*	0.21 (-0.11, 0.56)
mean headache intensity of scheduled recordings only	41.1 (22.4)*	0.75 (0.00, 0.93)	29.2 (16.2)*	0.16 (-0.09, 0.48)
mean headache intensity of event-contingent recordings only	60.3 (23.0)	0.81 (0.41, 0.95)	66.8 (12.2)	0.21 (-0.22, 0.60)
mean headache intensity of recordings when headaches were present	44.7 (19.0)*	0.77 (0.24, 0.92)	40.5 (13.0)*	0.29 (-0.11, 0.65)
maximal headache intensity of all recordings	71.5 (19.0)*	0.64 (-0.08, 0.89)	83.0 (11.7)*	0.23 (-0.10, 0.59)

ICC (A, 1), intraclass correlation coefficient of absolute agreement; SD, standard deviation; C.I., confidence interval.

* *P *< 0.001, vs. recalled headache intensity.

Recalled headache intensity was compared with some indices of momentary headache intensity.

High SD group is a group of patients whose headache intensity was highly variable.

Ecological momentary assessment (EMA) has been proposed as a reliable method to assess and record events and subjective symptoms as well as physiological and behavioral data in natural settings [9]. Recently, Burton et al. [10] reported an excellent systematic review of electronic diaries for self-report data such as pain and symptoms. In addition, Smyth and Stone [11] made a fabulous review of EMA research in behavioral medicine showing some examples including objective data such as cortisol in healthy people and peak expiratory flow in asthma patients. However, little attention has been paid to physical activity recorded objectively in the context of EMA research, although some studies using physical activity as an EMA variable have been published. Therefore, in the present brief review, we would like to introduce EMA and its usefulness, especially physical activity data as an object variable in EMA. In addition, we would like to discuss future applications of EMA for intervention in lifestyle-related physical diseases such as obesity and diabetes mellitus.

## Ecological momentary assessment (EMA)

EMA is a sampling method developed 'to assess phenomena at the moment they occur in natural settings, thus maximizing ecological validity while avoiding retrospective recall' [9]. When applying EMA to stress-related diseases such as IBS and asthma, paper-and-pencil diaries have often been used as recording devices [12,13]. However, such diaries have the disadvantage of 'faked compliance', i.e. disguise of compliance by recording data at times other than those designated even if signaling is used to remind patients of recording data [14-16].

To overcome this 'faked compliance', computerized EMA, i.e. EMA using computers as electronic diaries, has been developed. In computerized EMA, the input time is also recorded by the device in order to avoid faked compliance [17]. In addition, electronic diaries are able to issue randomly scheduled prompts to solicit data entry, thus reducing the risk that the assessment schedule may affect a natural rhythm in patients' lives [17].

**Figure 1:**
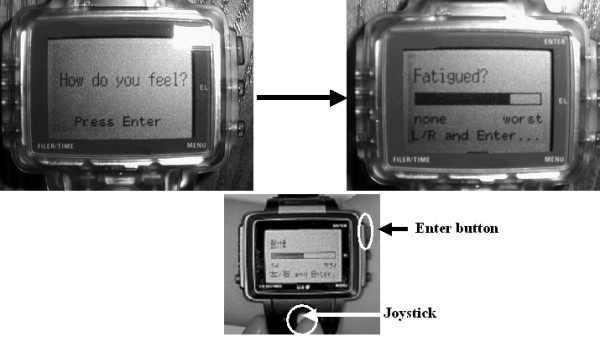
**The watch-type computer device used in previous studies **[8,20,55,57]. It is easy to manipulate the device using the joystick to lengthen or shorten the bar-like visual analogue scale and to push the enter-button to record the scale.

Electronic diaries have been often implemented in palm-size computers [18-20] or watch-type computers (Fig. 1) [8,21] while an electronic touch-tone telephone system has been also used as a validated system [22]. The details of proposed guidelines and designing protocols for EMA are beyond the scope of the present report and have been described in previous reports [17,23,24].

## Analysis of EMA data

Generally, the structure of momentary data is complex. Most time-series data tend to show serial autocorrelation, which violates the assumption of independence underlying parametric statistical methods such as multivariate regression. In addition, repeated measures of analysis of variance (RM-ANOVA), a well-known technique for analyzing data collected over time, cannot be applied to the analysis of most real-time data because the assumption underlying the strict data structure required by RM-ANOVA are mostly not met, which includes equally spaced data and no missing data. In contrast, multilevel modeling is able to deal with many characteristics of momentary data collected by EMA [25]. A comprehensive step-by-step lecture for analyzing real-time data using multilevel modeling was described in a previous report by Schwartz [26].

## Previous studies using EMA in stress-related diseases

### Assessing self-report symptoms

There have been many studies using EMA to assess subjective symptoms in stress-related physical diseases. Most studies assess pain in pain-related physical diseases such as rheumatoid arthritis, fibromyalgia and headaches [18-20,27-39]. Fatigue has also been evaluated using EMA in many studies [21,40-45] because pain and fatigue are difficult to assess objectively. Eating disorders are also major problems to be handled, and there have been some studies using EMA to assess symptoms in patients with bulimia nervosa or with binge eating disorder in natural settings [46-54] although there have been few studies on anorexia nervosa. Recently, attempts have been made to apply EMA to other stress-related physical diseases such as IBS [55].

### Assessing subjective symptoms and objective data using wearable devices

Recently, there have been some studies, but not many, using wearable devices for assessing and recording objective data as well as subjective symptoms in natural settings. Kamarck et al. [56] showed, using electronic diaries and ambulatory blood pressure monitoring, that daily psychosocial demands caused elevation of ambulatory blood pressure. Smyth et al. [57] reported the association between psychological stress and salivary cortisol secretion. Saito et al. [58] reported that chemical substances in the air caused subjective symptoms in patients with multiple chemical sensitivity using electronic diaries and wearable gas-samplers in natural settings. Affleck et al. [59] and Smyth et al. [60] reported that peak expiratory flow rate was associated with psychosocial factors in asthma patients using peak flow meters. In addition, there have been some recent studies [61-64] assessing the relationship between subjective symptoms and physical activity using actigraphy in natural settings. These previous studies using actigraphy have also successfully yielded many novel findings.

**Figure 2:**
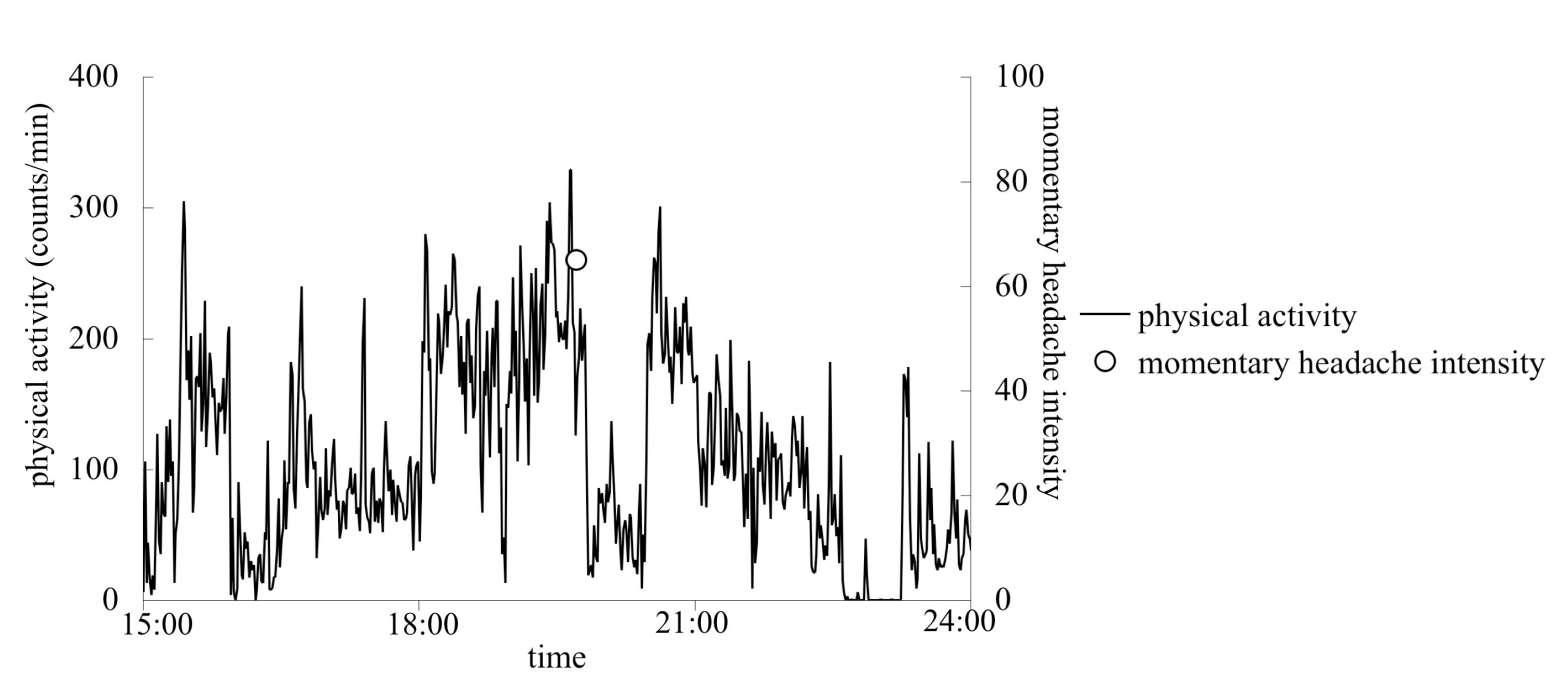
**Example of data for momentary headache intensity and physical activity of a patient with tension-type headache **[57]. Line graph shows physical activity counts per minute. Open circle shows momentary headache intensity. Headache was exacerbated and the patient added an event-contingent recording around 19:30 (open circle). It seems that physical activity was decreased after the headache exacerbation.

In our recent study [61], for example, watch-type wearable computers equipped with an actigraphy inside were used for recording momentary headache intensity and physical activity simultaneously (Figure 1). The results of the study showed objectively that there were significant negative associations between headache intensity and the simultaneous and subsequent activity level, and that activity level was significantly reduced at headache exacerbations (Figure 2). There have been few devices that are able to collect long-term objective data noninvasively in natural settings. Therefore, actigraphy is one of the most useful devices for EMA research at this point.

## Future direction

Because recent studies using actigraphy show significant findings by applying sophisticated time-series data analyses [65-68], more attention should be paid to objectively assessed and recorded data such as locomotor activity and behavior in natural settings. In addition, autonomic nervous function has been reported to be impaired in patients with psychosomatic disorders [69-71]. Therefore, in the near future, studies should be performed for longer duration, i.e. a few years [72], using wearable devices to simultaneously collect behavioral data, locomotor activity, and physiological data as well as subjective symptoms [73,74].

In addition, one future application of EMA is a tool for intervention in lifestyle-related physical diseases such as obesity and diabetes mellitus. In psychiatric diseases such as anxiety disorders, there have already been some studies [75-77] on computerized cognitive behavioral therapy (CCBT) using palm-size computers, although the efficacy of CCBT has not been confirmed. In addition, internet-based intervention has been reported to be effective in lifestyle-related diseases such as obesity [78,79] and diabetes [80]. However, there have been few studies on the effect of behavioral change programs or CCBT using palm-size computers, which could provide timely feedback or intervention in natural settings. Therefore, application of EMA technique to intervention in lifestyle-related diseases should be conducted in the near future.

## Conclusion

Computerized EMA will be able to yield more fruitful findings about the relationships between psychosocial factors and stress-related diseases when wearable devices are developed to assess and record more physiological and behavioral data in natural settings.

## List of abbreviations used

IBS: irritable bowel syndrome; EMA: ecological momentary assessment; CCBT: computerized cognitive behavior therapy.

## Competing interests

The authors declare that they have no competing interests.

## Authors' contributions

KY, YY, and AA wrote and approved the final manuscript.

## References

[B1] Nicholl BI, Halder SL, Macfarlane GJ, Thompson DG, O'brien S, Musleh M, McBeth J (2008). Psychosocial risk markers for new onset irritable bowel syndrome – Results of a large prospective population-based study. Pain.

[B2] Labbe EE, Murphy L, O'Brien C (1997). Psychosocial factors and prediction of headaches in college adults. Headache.

[B3] Wainwright NW, Surtees PG, Wareham NJ, Harrison BD (2007). Psychosocial factors and incident asthma hospital admissions in the EPIC-Norfolk cohort study. Allergy.

[B4] Bradburn NM, Rips LJ, Shevell SK (1987). Answering autobiographical questions: the impact of memory and inference on surveys. Science.

[B5] Hfford MR, Stone AA, Shiffman S, Atienza AA, Nebeling L (2007). Special methodological challenges and opportunities in ecological momentary assessment. The Science of REAL-TIME Data Capture.

[B6] Stone AA, Broderick JE, Shiffman SS, Schwartz JE (2004). Understanding recall of weekly pain from a momentary assessment perspective: absolute agreement, between- and within-person consistency, and judged change in weekly pain. Pain.

[B7] Stone AA, Schwartz JE, Broderick JE, Shiffman SS (2005). Variability of momentary pain predicts recall of weekly pain: a consequence of the peak (or salience) memory heuristic. Pers Soc Psychol Bull.

[B8] Kikuchi H, Yoshiuchi K, Miyasaka N, Ohashi K, Yamamoto Y, Kumano H, Kuboki T, Akabayashi A (2006). Reliability of recalled self-report on headache intensity: investigation using ecological momentary assessment technique. Cephalalgia.

[B9] Stone AA, Shiffman S (1994). Ecological momentary assessment (EMA) in behavioral medicine. Ann Behav Med.

[B10] Burton C, Weller D, Sharpe M (2007). Are electronic diaries useful for symptom research? A systematic review. J Psychosom Res.

[B11] Smyth JM, Stone AA (2003). Ecological momentary assessment research in behavioral medicine. J Happiness Stud.

[B12] Cain KC, Headstrom P, Jarrett ME, Motzer SA, Park H, Burr RL, Surawicz CM, Heitkemper MM (2006). Abdominal pain impacts quality of life in women with irritable bowel syndrome. Am J Gastroenterol.

[B13] Johnston SL, Blasi F, Black PN, Martin RJ, Farrell DJ, Nieman RB, TELICAST Investigators (2006). The effect of telithromycin in acute exacerbations of asthma. N Engl J Med.

[B14] Stone AA, Shiffman S, Schwartz JE, Broderick JE, Hufford MR (2002). Patient non-compliance with paper diaries. Br Med J.

[B15] Broderick JE, Schwartz JE, Shiffman S, Hufford MR, Stone AA (2003). Signaling does not adequately improve diary compliance. Ann Behav Med.

[B16] Burke LE, Sereika SM, Music E, Warziski M, Styn MA, Stone A (2008). Using instrumented paper diaries to document self-monitoring patterns in weight loss. Contemp Clin Trials.

[B17] Stone AA, Shiffman S (2002). Capturing momentary, self-report data: a proposal for reporting guidelines. Ann Behav Med.

[B18] Litcher-Kelly L, Stone AA, Broderick JE, Schwartz JE (2004). Associations among pain intensity, sensory characteristics, affective qualities, and activity limitations in patients with chronic pain: a momentary, within-person perspective. J Pain.

[B19] Turner JA, Mancl L, Aaron LA (2004). Pain-related catastrophizing: a daily process study. Pain.

[B20] Aaron LA, Turner JA, Mancl L, Brister H, Sawchuk CN (2005). Electronic diary assessment of pain-related variables: is reactivity a problem?. J Pain.

[B21] Yoshiuchi K, Cook DB, Ohashi K, Yamamoto Y, Kumano H, Kuboki T, Natelson BH (2007). A real-time assessment of the effect of exercise in chronic fatigue syndrome. Physiol Behav.

[B22] Camilleri M, Northcutt AR, Kong S, Dukes GE, McSorley D, Mangel AW (2000). Efficacy and safety of alosetron in women with irritable bowel syndrome: a randomised, placebo-controlled trial. Lancet.

[B23] Shiffman S, Stone AA, Shiffman S, Atienza AA, Nebeling L (2007). Designing protocols for ecological momentary assessment. The Science of REAL-TIME Data Capture.

[B24] Palm blad M, Tiplady B (2004). Electronic diaries and quesitonnaires: designing used interfaces that are easy for all patients to use. Qual Life Res.

[B25] Schwartz JE, Stone AA (1998). Strategies for analyzing ecological momentary assessment data. Health Psychol.

[B26] Schhwartz JE, Stone AA, Shiffman S, Atienza AA, Nebeling L (2007). The analysis of real-time momentary data. The Science of REAL-TIME Data Capture.

[B27] Aaron LA, Mancl L, Turner JA, Sawchuk CN, Klein KM (2004). Reasons for missing interviews in the daily electronic assessment of pain, mood, and stress. Pain.

[B28] Turner JA, Mancl L, Aaron LA (2005). Brief cognitive?behavioral therapy for temporomandibular disorder pain: effects on daily electronic outcome and process measures. Pain.

[B29] Stone AA, Broderick JE, Schwartz JE, Shiffman S, Litcher-Kelly L, Calvanese P (2003). Intensive momentary reporting of pain with an electronic diary: reactivity, compliance, and patient satisfaction. Pain.

[B30] Litt MD, Shafer D, Napolitano C (2004). Momentary mood and coping processes in TMD pain. Health Psychol.

[B31] Roelofs J, Peters ML, Patijn J, Schouten EG, Vlaeyen JW (2004). Electronic diary assessment of pain-related fear, attention to pain, and pain intensity in chronic low back pain patients. Pain.

[B32] Giffin NJ, Ruggiero L, Lipton RB, Silberstein SD, Tvedskov JF, Olesen J, Altman J, Goadsby PJ, Macrae A (2003). Premonitory symptoms in migraine: an electronic diary study. Neurology.

[B33] Affleck G, Urrows S, Tennen H, Higgins P, Abeles M (1996). Sequential daily relations of sleep, pain intensity, and attention to pain among women with fibromyalgia. Pain.

[B34] Affleck G, Tennen H, Zautra A, Urrows S, Abeles M, Karoly P (2001). Women's pursuit of personal goals in daily life with fibromyalgia: a value-expectancy analysis. J Consult Clin Psychol.

[B35] Zautra A, Smith B, Affleck G, Tennen H (2001). Examinations of chronic pain and affect relationships: applications of a dynamic model of affect. J Consult Clin Psychol.

[B36] Peters ML, Sorbi MJ, Kruise DA, Kerssens JJ, Verhaak PF, Bensing JM (2000). Electronic diary assessment of pain, disability and psychological adaptation in patients differing in duration of pain. Pain.

[B37] Honkoop PC, Sorbi MJ, Godaert GL, Spierings EL (1999). High-density assessment of the IHS classification criteria for migraine without aura: a prospective study. Cephalalgia.

[B38] Viane I, Crombez G, Eccleston C, Devulder J, De Corte W (2004). Acceptance of the unpleasant reality of chronic pain: effects upon attention to pain and engagement with daily activities. Pain.

[B39] Stone AA, Broderick JE, Porter LS, Kaell AT (1997). The experience of rheumatoid arthritis pain and fatigue: examining momentary reports and correlates over one week. Arthritis Care Res.

[B40] Buysse DJ, Thompson W, Scott J, Franzen PL, Germain A, Hall M, Moul DE, Nofzinger EA, Kupfer DJ (2007). Daytime symptoms in primary insomnia: a prospective analysis using ecological momentary assessment. Sleep Med.

[B41] Hacker ED, Ferrans CE (2007). Ecological momentary assessment of fatigue in patients receiving intensive cancer therapy. J Pain Symptom Manage.

[B42] Curran SL, Beacham AO, Andrykowski MA (2004). Ecological momentary assessment of fatigue following breast cancer treatment. J Behav Med.

[B43] Sonnenschein M, Sorbi MJ, van Doornen LJ, Schaufeli WB, Maas CJ (2007). Evidence that impaired sleep recovery may complicate burnout improvement independently of depressive mood. J Psychosom Res.

[B44] Sonnenschein M, Mommersteeg PM, Houtveen JH, Sorbi MJ, Schaufeli WB, van Doornen LJ (2007). Exhaustion and endocrine functioning in clinical burnout: an in-depth study using the experience sampling method. Biol Psychol.

[B45] Friedberg F, Quick J (2007). Alexithymia in chronic fatigue syndrome: associations with momentary, recall, and retrospective measures of somatic complaints and emotions. Psychosom Med.

[B46] Wild B, Eichler M, Feiler S, Friederich HC, Hartmann M, Herzog W, Zipfel S (2007). Dynamic analysis of electronic diary data of obese patients with and without binge eating disorder. Psychother Psychosom.

[B47] Hilbert A, Tuschen-Caffier B (2007). Maintenance of binge eating through negative mood: a naturalistic comparison of binge eating disorder and bulimia nervosa. Int J Eat Disord.

[B48] Wonderlich SA, Crosby RD, Engel SG, Mitchell JE, Smyth J, Miltenberger R (2007). Personality-based clusters in bulimia nervosa: differences in clinical variables and ecological momentary assessment. J Personal Disord.

[B49] Wonderlich SA, Rosenfeldt S, Crosby RD, Mitchell JE, Engel SG, Smyth J, Miltenberger R (2007). The effects of childhood trauma on daily mood lability and comorbid psychopathology in bulimia nervosa. J Trauma Stress.

[B50] Vansteelandt K, Rijmen F, Pieters G, Probst M, Vanderlinden J (2007). Drive for thinness, affect regulation and physical activity in eating disorders: a daily life study. Behav Res Ther.

[B51] Boseck JJ, Engel SG, Allison KC, Crosby RD, Mitchell JE, de Zwaan M (2007). The application of ecological momentary assessment to the study of night eating. Int J Eat Disord.

[B52] Stein RI, Kenardy J, Wiseman CV, Dounchis JZ, Arnow BA, Wilfley DE (2007). What's driving the binge in binge eating disorder?: A prospective examination of precursors and consequences. Int J Eat Disord.

[B53] Engel SG, Boseck JJ, Crosby RD, Wonderlich SA, Mitchell JE, Smyth J, Miltenberger R, Steiger H (2007). The relationship of momentary anger and impulsivity to bulimic behavior. Behav Res Ther.

[B54] Wegner KE, Smyth JM, Crosby RD, Wittrock D, Wonderlich SA, Mitchell JE (2002). An evaluation of the relationship between mood and binge eating in the natural environment using ecological momentary assessment. Int J Eat Disord.

[B55] Kajander K, Latti M, Hatakka K, Korpela R (2007). An electronic diary versus a paper diary in measuring gastrointestinal symptoms. Dig Liver Dis.

[B56] Kamarck TW, Janicko DL, Shiffman S, Polk DE, Muldoon MF, Liebenauer LL, Schwartz JE (2002). Psychosocial demands and ambulatory pressure: a field assessment approach. Physiol Behav.

[B57] Smyth J, Ockenfels M, Porter L, Kirschbaum C, Hellhammer D, Stone A (1998). The association between daily stressors, mood and salivary cortisol secretion. Psychoneuroendocrinology.

[B58] Saito M, Kumano H, Yoshiuchi K, Kokubo N, Ohashi K, Yamamoto Y, Shinohara N, Yanagisawa Y, Sakabe K, Miyata M, Ishikawa S, Kuboki T (2005). Symptom Profile of Multiple Chemical Sensitivity in Actual Life. Psychosom Med.

[B59] Affleck G, Apter A, Tennen H, Reisine S, Barrows E, Willard A, Unger J, ZuWallack R (2000). Mood states associated with transitory changes in asthma symptoms and peak expiratory flow. Psychosom Med.

[B60] Smyth J, Soefer M, Hurewitz A, Kliment A, Stone A (1999). Daily psychosocial factors predict levels and diurnal cycles of asthma symptomatology and peak flow. J Behav Med.

[B61] Kikuchi H, Yoshiuchi K, Ohashi K, Yamamoto Y, Akabayashi A (2007). Tension-type headache and physical activity: an actigraphic study. Cephalalgia.

[B62] Liszka-Hackzell JJ, Martin DP (2004). An analysis of the relationship between activity and pain in chronic and acute low back pain. Anesth Analg.

[B63] Kop WJ, Lyden A, Berlin AA, Ambrose K, Olsen C, Gracely RH, Williams DA, Clauw DJ (2005). Ambulatory monitoring of physical activity and symptoms in fibromyalgia and chronic fatigue syndrome. Arthritis Rheum.

[B64] Bazelmans E, Bleijenberg G, Voeten MJ, Meer JW van der, Folgering H (2005). Impact of a maximal exercise test on symptoms and activity in chronic fatigue syndrome. J Psychosom Res.

[B65] Ohashi K, Yamamoto Y, Natelson BH (2002). Activity rhythm degrades after strenuous exercise in chronic fatigue syndrome. Physiol Behav.

[B66] Yoshiuchi K, Yamamoto Y, Niwamoto H, Watsuji T, Kumano H, Kuboki T (2003). Behavioral power-law exponents in the usage of electric appliances correlate mood states in the elderly. Int J Sport Health Sci.

[B67] Ohashi K, Bleijenberg G, Werf S van der, Prins J, Amaral LA, Natelson BH, Yamamoto Y (2004). Decreased fractal correlation in diurnal physical activity in chronic fatigue syndrome. Methods Inform Med.

[B68] Nakamura T, Kiyono K, Yoshiuchi K, Nakahara R, Struzik ZR, Yamamoto Y (2007). Universal scaling law in human behavioral organization. Phys Rev Lett.

[B69] Yamamoto Y, LaManca JJ, Natelson BH (2003). A measure of heart rate variability is sensitive to orthostatic challenge in women with chronic fatigue syndrome. Exp Biol Med.

[B70] Peckerman A, LaManca JJ, Qureishi B, Dahl KA, Golfetti R, Yamamoto Y, Natelson BH (2003). Baroreceptor reflex and integrative stress responses in chronic fatigue syndrome. Psychosom Med.

[B71] Yoshiuchi K, Quigley KS, Ohashi K, Yamamoto Y, Natelson BH (2004). Use of time-frequency analysis to investigate temporal patterns of cardiac autonomic response during head-up tilt in chronic fatigue syndrome. Auton Neurosci.

[B72] Yoshiuchi K, Nakahara R, Kumano H, Kuboki T, Togo F, Watanabe E, Yasunaga A, Park MH, Shephard RJ, Aoyagi Y (2006). Yearlong physical activity and depressive symptoms in older Japanese adults: cross-sectional data from the Nakanojo Study. Am J Geriatr Psychiatry.

[B73] Aoyagi N, Ohashi K, Tomono S, Yamamoto Y (2000). Temporal contribution of body movement to very long-term heart rate variability in humans. Am J Physiol Heart Circ Physiol.

[B74] Struzik ZR, Yoshiuchi K, Sone M, Ishikawa T, Kikuchi H, Kumano H, Watsuji T, Natelson BH, Yamamoto Y (2007). "Mobile Nurse" platform for ubiquitous medicine. Methods Inform Med.

[B75] Newman MG, Kenardy J, Herman S, Taylor CB (1997). Comparison of palmtop-computer-assited brief cognitive-behavioral treatment to cognitive-behavioral treatment for panic disorder. J Consult Clin Psychol.

[B76] Newman MG, Consoli AJ, Taylor CB (1999). A palmtop computer program for the treatment of generalized anxiety disorder. Behav Modif.

[B77] Przeworski A, Newman MG (2004). Palmtop computer-assisted group therapy for social phobia. J Clin Psychol.

[B78] Tate DG, Wing RR, Winett RA (2001). Using Internet technology to deliver a behavioral weight loss program. JAMA.

[B79] Harvey-Berino J, Pintauro S, Buzzell P, Gold EC (2004). Effect of Internet support on the long-term maintenance of weight loss. Obes Res.

[B80] Tate DF, Jackvony EH, Wing RR (2003). Effects of Internet behavioral counselling on weight loss in adults at risk for Type 2 diabetes. JAMA.

